# Wearable System Applications in Performance Analysis of RaceRunning Athletes with Disabilities

**DOI:** 10.3390/s24247923

**Published:** 2024-12-11

**Authors:** Mohsen Shafizadeh, Keith Davids

**Affiliations:** School of Sport and Physical Activity, College of Health, Wellbeing and Life Sciences, Sheffield Hallam University, Sheffield S10 2BP, UK; k.davids@shu.ac.uk

**Keywords:** RaceRunning, adaptations, training loads, wearable-motion sensors, performance efficiency, feedback

## Abstract

RaceRunning is a sport for disabled people and successful performance depends on reducing the amount of time spent travelling a specific distance. Performance analysis in RaceRunning athletes is based on traditional methods such as recording race time, distances travelled and frequency (sets and reps) that are not sufficient for monitoring training loads. The aims of this study were to monitor training loads in typical training sessions and evaluate technical adaptations in RaceRunning performance by acquiring sensor metrics. Five elite and competitive RaceRunning athletes (18.2 ± 2.3 yrs) at RR2 and RR3 levels were monitored for 8 weeks, performing in their usual training sessions while wearing unobtrusive motion sensors. The motion sensors were attached to the waist and lower leg in all training sessions, each lasting between 80 and 90 min. Performance metrics data collected from the motion sensors included player loads, race loads, work/rest ratio and impact shock directions, along with training factors (duration, frequency, distance, race time and rest time). Results showed that weekly training loads (player and race loads) followed acceptable threshold levels, according to assessment criteria (smallest worthwhile change, acute/chronic work ratio). The relationship between race velocity (performance index) and race load was non-linear and statistically significant, which led to different performance efficiency groups. Wearable motion sensor metrics revealed small to moderate technical adaptations following repeated sprint attempts in temporal running performance, variability and consistency. In conclusion, using a wearable-based system is an effective feedback tool to monitor training quality, revealing important insights into adaptations to training volumes in disabled athletes.

## 1. Introduction

The number of disabled people who participate in sports is growing because of the noted positive impact of sports participation on wellbeing, health-related fitness components and motivation [1]. RaceRunning is a para sport: an adapted form of running for disabled people requiring use of a three-wheeled bike during locomotion. Due to its ergonomic design, it provides an opportunity for disabled people to move, despite severe limb impairments [2]. RaceRunning is a competitive track event for people with severe coordination impairments [3] and was registered as a para sport in 2017, initially for people with brain injuries, later including cerebral palsy (CP) patients [4]. Due to the inclusive nature of this sport for different groups of disabled individuals, it can provide valuable opportunities for increasing the participation of disabled people in organised physical activity for their health and wellbeing and also for competitive purposes.

Applications of technology in modern sports have become more common, due to their impact on performance. Nowadays, many team and individual sports benefit from augmented informational feedback that is provided by technologies to enhance performance, monitor training volumes, prevent injuries and evaluate the effectiveness of practice on skills and physical fitness [5]. For some time, performance analysis in sport, using technologies such as video analysis systems and wearable sensors, has been an important element of the coaching process [6]. The significant roles of the support technology have been appreciated within the cycle of competition, reflection, decision-making and performance preparation [7] by coaching and sport scientists.

The role of technology in practice and competition for people with disabilities is paramount, too. In a systematic review of 39 studies [8], the importance of wearable technologies for monitoring practice and activity in disabled athletes has been emphasised. Results revealed that the two common technologies used for performance assessment were motion sensors and portable electromyography (EMG) systems. The authors categorised their applications into four distinct groups: athlete classification, injury prevention, performance characterisation/training optimisation and equipment customisation. Whilst the selected sports were mainly wheelchair sports (rugby, basketball, racing and curling), there were three studies in running and one study in RaceRunning and in people with CP that indicated a lack of available evidence in RaceRunning sport.

Using wearable systems for assessment of running, jogging and sprinting performance in both practice and competition is common [9]. For example, wearable systems provide valuable information on physical exertion and movement economy, evaluated according to travelled distance, velocity, acceleration and deceleration profiles, applicable for planning strength and conditioning programmes. The wearable motion sensors that are used frequently in sports are inertial measurement units (IMUs) and global tracking systems (GPS). They are integrated into different hardware components (accelerometer, gyroscope, magnetometer) in a small case, using a conventional metric, player load (a converted form of three-axis acceleration signals), to assess acute (daily time scale) and chronic (aggregated weekly and monthly time scales) workloads. These sensors have been used for monitoring training workload and prediction of overuse injuries in sports such as rugby [10], football [11], volleyball [12] and swimming [13].

Despite the popularity of running in different sports, running-related injuries are still prevalent [14] due to an imbalance between training and recovery [15]. One way to estimate workloads and predict risk of overuse injuries in runners is by using wearable motion sensors. Reports from different cohorts of runners have revealed that such systems are feasible and informative for guiding training sessions, because of their handy size and the meaningfulness of data analytics platforms to monitor performance over time [16,17,18]. For example, Cloosterman et al. [17] showed that GPS data were functional in calculating weekly acute-to-chronic workload ratio (ACWR) and associations between training load and onset of running-related knee injuries in recreational runners. ACWR is a useful metric to calculate an athlete’s ability to tolerate sudden changes in load. It also is a valid predictor of risk of overuse injuries in sports. For example, in rugby players, an ACWR value above 2.0 predicted the likelihood of injuries [19]. Neal et al. [16] reported 70% adherence and 92% successful data collection in recreational runners through using a wrist IMU/GPS sensor in monitoring acute training loads for prediction of injury.

One concept relevant to sports injuries is training adaptation, which indicates the body’s response to training stress [20]. To achieve optimal individual performance in running, coaches usually manipulate some training factors related to external loads, such as intensity, frequency, duration, distance and number of repetitions or training volume [21]. Adding other training metrics, such as external or internal workload metrics (e.g., cumulative shock, rating of perceived exertion), to the conventional training methods of runners could provide valuable information to individualise training adaptations and potentially reduce the risk of overtraining and overuse injuries [22].

The number of studies that have used wearable motion sensors in monitoring training loads in disabled athletes for performance enhancement or injury prevention is limited. Fulton et al. [23] used IMU sensors in monitoring Paralympic swimmers to investigate the role of kicking in freestyle swimming, by quantifying variables like kick count, rate and amplitude. To investigate changes in training load according to a specific athlete’s activity, some studies have used motion sensors on the wheelchair frame to obtain data on performance parameters such as mean linear acceleration, rotational velocity and acceleration in wheelchair basketball [24] and wheelchair tennis [25] or for computation of energy expenditure and intensity level of players in wheelchair rugby [26]. Furthermore, heart rate sensors have been used to monitor training load in running [27,28] and wheelchair basketball [24].

There is no evidence to suggest that the training workloads in RaceRunning athletes need to be the same as those of able-bodied individuals, except through use of conventional methods of recording indirect training variables (number of races undertaken, time, distance, etc.). This method also is not adequate to gain real-time data on the body’s responses to training stress (volume). Hence, using wearable sensors to collect more information about body impact shock (running loads) could help coaches and trainers to optimise training programmes based on training feedback for enhancing performance and reducing risks of overtraining and overuse injuries, specifically in disabled athletes who often have structural and functional variations to contend with. Thus, the aims of this study were to monitor training loads in typical training sessions, and evaluate technical adaptations in RaceRunning performance from sensor metrics.

## 2. Methods

### 2.1. Participants

The study used a descriptive/prospective design in which the training status of the participants was recorded without any intervention. Five (two male and three female) elite and competitive athletes (age: 18.2 ± 2.3 years; body mass: 51.21 ± 5.4 kg; and height: 167.1 ± 6.5 cm) were non-randomly selected from a local RaceRunning club. Because of the purpose of the study and its descriptive nature, all members of the club were recruited non-randomly. The eligibility criteria were disabled athletes at levels of RR2 and RR3 RaceRunning levels (RR2: n = 2 and RR3: n = 3), according to CP International Sports and Recreation Association classifications. Athletes in the RR2 class have spasticity, athetosis, ataxia dystonia, or muscle weakness, which limit the effective pushing movements of the lower extremities. Athletes in the RR3 class have mild to moderate involvement in one or both upper extremities, fair to good trunk control, and moderate involvement of the lower extremities. Other eligibility criteria were long-term neurological conditions, including spastic cerebral palsy (n = 4) and acquired brain injuries (n = 1), freedom from any musculoskeletal injury during data collection and participation in competitions (mean experience: 3.0 ± 0.7 years).

Their level of ambulation was assessed using the Functional Mobility Scale [29], in which they were assessed on their perceived ability to walk different distances (5 m, 50 m, 500 m) independently (rate = 6) to using a wheelchair (rate = 1). The participants rated their ability at 6 in the 5 m distance and at 1 in the 500 m distance. Participants completed a consent form in the presence of their carers. The study was approved by an institutional University research ethics committee and conducted according to the ethical guidelines of the Helsinki Declaration of 1964.

### 2.2. Materials

The main components of the performance analysis system in this study were 9-axis (3-axis Accelerometer, 3-axis Gyroscope, 3-axis Magnometer) and low-mass (<3 g) wearable motion sensors (MetaMotion R, MBIENT LAB Co., San Jose, CA, USA). The sensors were equipped with Bosch Sensortec (Stuttgart, Germany), which combines measurements of the accelerometer, gyroscope and magnetometer to provide a robust calculation of the orientation vector (3-axis Euler angle).

The 2 motion sensors were used throughout the performance analysis period for capturing training loads on different body parts. A waist sensor was attached to the low-back area (L2–L3) for measuring whole body training load, and a leg sensor was attached to the medial-distal part of the right tibia for measuring lower-limb impact shock as well as recognising running phases (stance, flight and stride). For detecting running phases, the gyroscope and accelerometer of the tibia sensor were synchronised. The tibia sensor has previously been validated for use in different activities [30]. The sensors were secured by double-sided tape and Velcro adjustable straps (Presco, Swindon, UK). Motion sensor orientation was calibrated by the sensor–body alignment. The tibia sensor was placed so that the X axis was aligned with the shank length in the standing position (X: superior–inferior; Y: anterior–posterior; Z: mediolateral). The waist sensor alignment was 90 degrees rotation relative to the tibia sensor (X: mediolateral; Y: superior–inferior; Z: anterior–posterior). The sensor’s sample rate was set at a frequency of 400 Hz.

The motion sensors were programmed by a free mobile application (MetaBase, MBIENT LAB, Co., San Jose, CA, USA). MetaBase is a user-friendly application that runs on both iOS and Android platforms. This application can synchronise sensors for simultaneous data capturing, saving and exporting. In addition, it was possible to customise data collection in terms of signal type (acceleration, gyroscope, etc.), speed (25 Hz to 800 Hz) and transmission mode (streaming, logging). For this study, all sessions were recorded through the logging mode, and raw data were exported as a CSV file for further analysis.

A Polar Heart Rate sensor (Polar Sense armband and chest strap) was used to monitor internal load during the training session. The Polar armband is an optical sensor that was wrapped around the right upper arm and connected via Bluetooth to the Polar mobile application (Polar Flow App, version 6.24.0) for recording the heart rate per athlete.

### 2.3. Procedure

**Figure 1:**
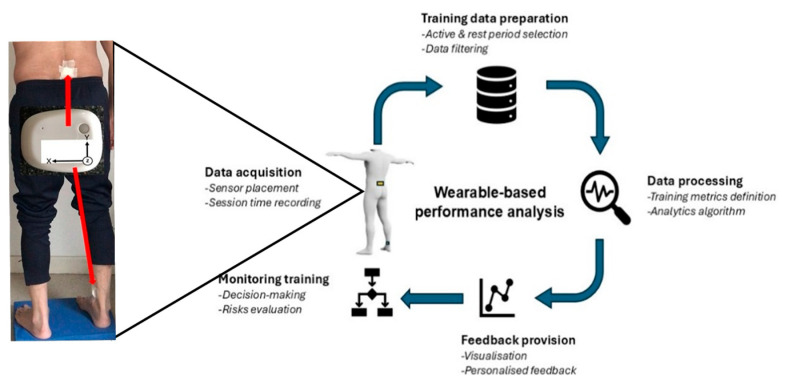
Diagram of data management procedure in using the wearable system for monitoring RaceRunning performance. The main aims (bold) and tasks (italic) from session data collection to feedback provision are presented.

The data collection protocol was followed according to Figure 1. The principal investigator (MS) was a performance analyst in the RaceRunning club who worked with the participants and the coaching team to discuss the protocol. Some stages of the protocol, such as data collection, feedback provision and training monitoring (see Figure 1), required effective communication with the coach and athletes to enhance the viability and feasibility of the wearable-based system in the field. Data collection took place at an indoor athletics track where the participants trained for one day per week. Participants wore standard running shoes and clothing, and everyone had to use a RaceRunner bike (Petra Cross Runner, Quest 88 Ltd., Shifnal, UK) which was adjustable in terms of body dimensions. The coach supervised the training session, which consisted of a routine programme including warming up with stretching, low-velocity running and a main part that was planned based on the seasonal training volume in terms of the number of runs, distance and intensity. Sprint running occurred on a straight line track, ranging between 20 m and 100 m in distance. Usually, the sprint running (activity) period was followed by a rest period of 7–8 min for a full recovery. The rest periods were dynamic and included slow walking and active stretching.

The standard procedure for monitoring training volumes was the pen-paper method (using a training log notebook) in which the coach wrote the number of runs, their distances and the race time for each participant. The wearable-based performance analysis system was added to the traditional methods in this study, providing an objective assessment tool in sprint performance for assessing training loads and race intensity, and as a monitoring system to individualise optimal loads and prevent any risk of overtraining. The motion sensors were attached to the participant’s body before the start of the main training component and were removed after the cooling down period. In addition, the principal investigator recorded the start and the end of the training session to match it with the sensor timestamp (year/month/date/time). Each session lasted between 80 and 90 min, and the length of this study was 2 months.

The coaches and athletes were regularly provided group and individual delayed feedback (1 week) on the quality of training sessions, based on the defined training metrics/key performance indicators (KPIs) in RaceRunning (see the next section). The type of feedback was mainly provided as visual feedback in Excel charts. Over time, session-by-session variations and fluctuations in KPIs were provided in PowerPoint slides to facilitate strategic decision-making in the training plan.

### 2.4. Data Analysis

The wearable motion sensors provided different metrics in the raw data. However, KPIs were individualised and selected because they directly related to each athlete’s physical performance, reflecting the required workload, being indicative of body condition. Individualised KPIs were divided into 2 main groups: KPIs for training load monitoring and KPIs for technical adaptations.

*Training load KPIs:* Training loads were further divided into internal loads (HR) and external loads (travelled distance, player loads and race loads). Values of HR were captured directly from the Polar armband as beats/min and reported as maximum, minimum and average HR per session. External loads were mainly extracted from the acceleration of the waist sensor (XYZ) for the whole training session (player load) or sprint race (race load). The race load was different from the performer load in terms of excluding the rest periods. The loads were presented as total load or load per minute/seconds.

The extracted load from the 3-axis acceleration was defined by the method in sports performance in which the resultant acceleration was scaled by 100 [31]:

$$Player\;load=\sqrt{\left(ax1-ax-1\right)+\left(ay1-ay-1\right)+\left(az1-az-1\right)/100}$$

*a_x_ a_y_ a_z_* is acceleration in 3 axes.

Race time was recorded with a digital stopwatch by the same person every time and presented as seconds/milliseconds. Sprint speed was calculated by dividing distance run by race time for each participant.

To assess the quality of training and avoid overtraining, weekly changes in the race load and training load were evaluated by the Smallest Worthwhile Change (SWC) and ACWR methods.

The SWC was calculated as follows:

(1) SWC = 0.3 × SD_weekly change_
(2) Mean_weekly change_
(3) Upper and lower limits = Mean_weekly change_ ± SWC

The ACWR method was based on the ratio of session load for each performer divided by the average load (2-month average).

The above equations were used for the group data. If the individual weekly change was inside the group’s upper and lower limits, then it was defined as in the normal zone.

*Technical adaptation KPIs*: The technical body adaptations were calculated based on the changes in different aspects of the running cycle (stance, flight, foot placement) as the training session progressed and the impact shock directions (forward, sideways and upward) generally. The gyroscope of the leg sensor was used to assess the running cycle through a method used in previous studies [2,30]. In this method, the initial contact and toe-off points were detected when the angular velocity of the tibia (in degrees per second) reached its minimum value on the x-axis. Thus, stride time was determined by the time from the first initial contact moment to the next initial contact moment. Stance time was determined from the initial contact to the toe-off moments, and flight time was determined from the toe-off to the initial contact moments. This process was completed by using a custom-written MATLAB 9.2 program (MathWorks, Inc., Natick, MA, USA). The temporal pattern of the running cycle was assessed by using the average (mean), variability (coefficient of variation) and consistency/regularity (permutation entropy) values. The permutation entropy (PE) is a mathematical method for measuring the complexity of a time series [32]. In this study, we used PE for assessing the consistency of stride time in 100-m races through order equal to 3 (three strides in a row). If the 3-number units have the same order (e.g., [1,2,3]) each time, we assume that the time series has high predictability and low complexity (closer to PE = 0).

Different descriptive and inferential statistics were used to address the aims of this study. The main descriptive statistical methods calculated values of central tendency and variability, percentage and ratio using scatter diagrams. To monitor training loads in terms of weekly changes and their impact on sprint performance, a nonlinear regression test was used. Technical adaptations in running performance and impact load directions, due to training status, were tested with Wilcoxon-ranked order and Friedman tests, respectively. All methods were tested at a 95% confidence interval (two-tailed). We also calculated Cohen’s *d* effect size (ES) as an index of sensitivity of wearable sensors to detect the magnitude of the changes. The *d* values equal to 0.2, 0.5 and greater than 0.8 were classified as small, medium and large effects, respectively.

## 3. Results

### 3.1. Training Load Monitoring

**Figure 2:**
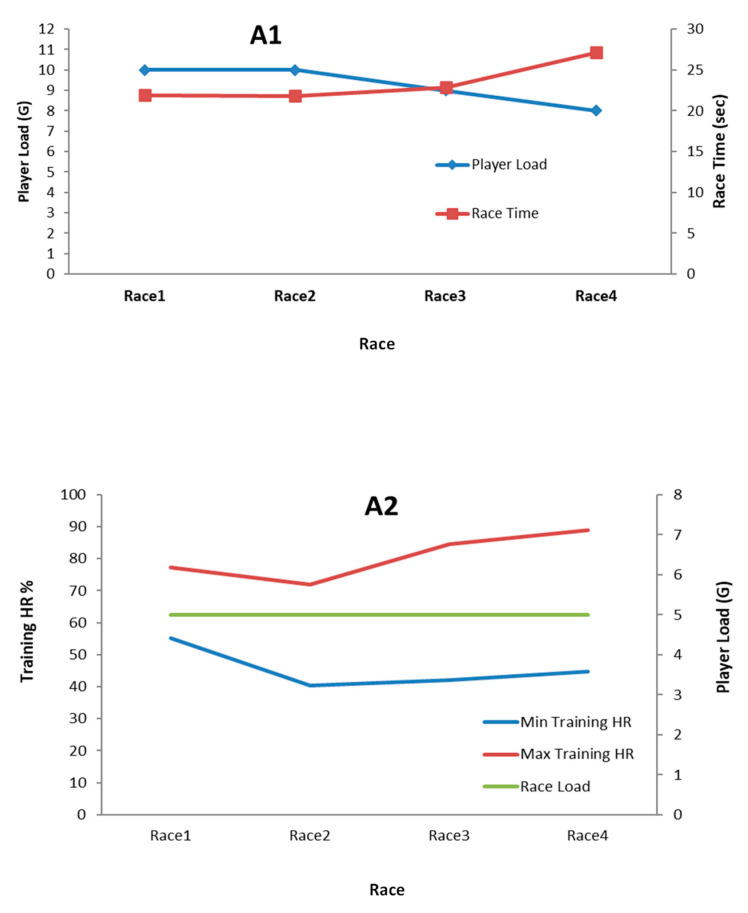
Examples of session training load monitoring procedures. Each athlete (A1,A2) ran multiple times as part of the training plan with enough rest periods. The main KPIs that were extracted in each session were related to training loads (external and internal) and sprint performance (time). We presented feedback based on the session performance individually and focused on the related KPIs. The performer load represents external load, whereas HR (beats/min) represents the internal load.

Presenting race load as an additional performance metric, along with race time (sprint performance), was informative to understand the relationship between the amount of workload and optimal individual performance. In other words, visualising the athlete’s body responses (HR) as internal load, relative to external load and sprint performance, together was useful informational feedback to adjust the activity/rest periods appropriately (see Figure 2).

**Figure 3:**
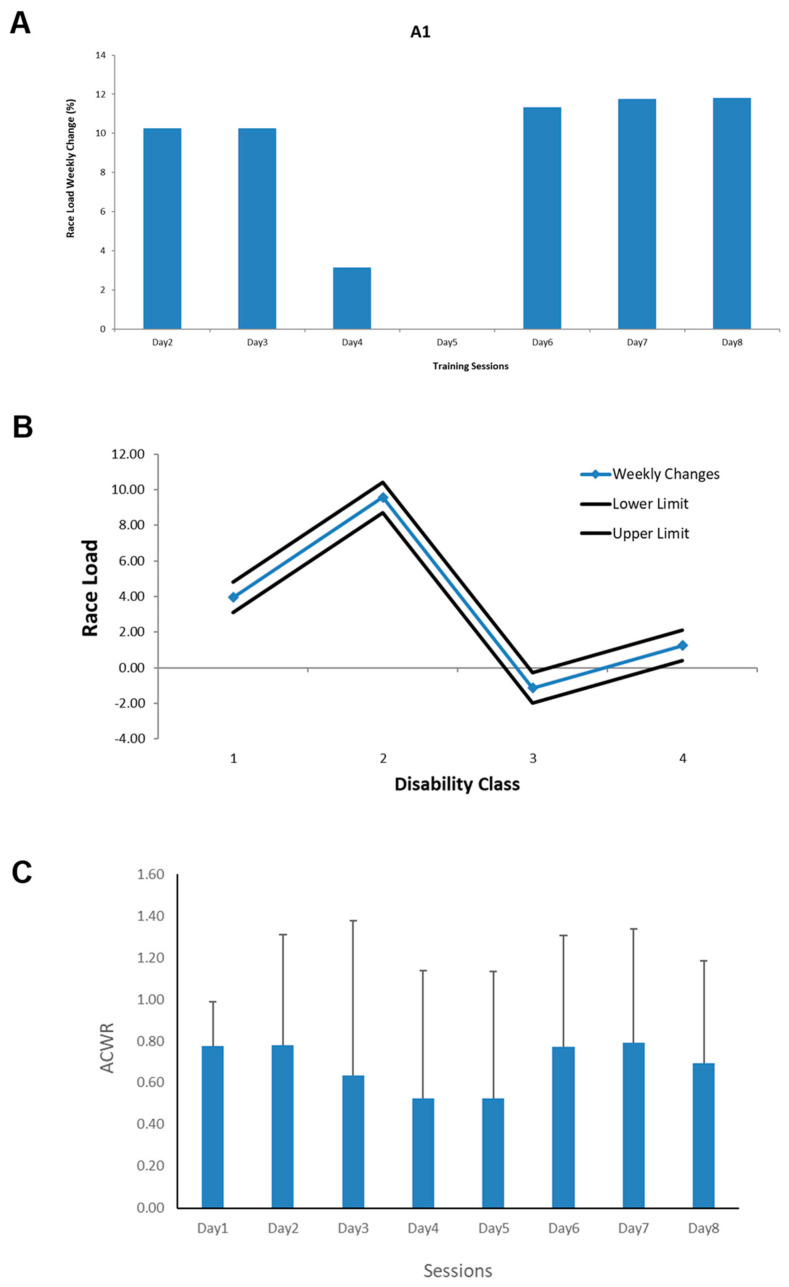
Monitoring weekly changes in training loads in one athlete (**A**). The descriptive changes in training load (**A**) were accompanied by two other criteria for checking the acceptable thresholds of training loads: SWC and ACWR. The results of AWC in different disability groups (**B**) and ACWR (mean ± SD) in different sessions (**C**) are presented in this Figure.

The weekly training load followed the acceptable threshold limits in all athletes, regardless of their disability levels (see Figure 3). The average change in weekly race load in some athletes (disability class 2) increased by 9.58%, whereas in disability class 3, the race load decreased by −1.13%. The discrepancy among the athletes’ race loads indicates the intra-individual variability in their responses to the same race distance. The ACWR displayed fluctuations on a weekly basis (0.52–0.79). The average group ACWR was 0.69 (±0.16).

**Figure 4:**
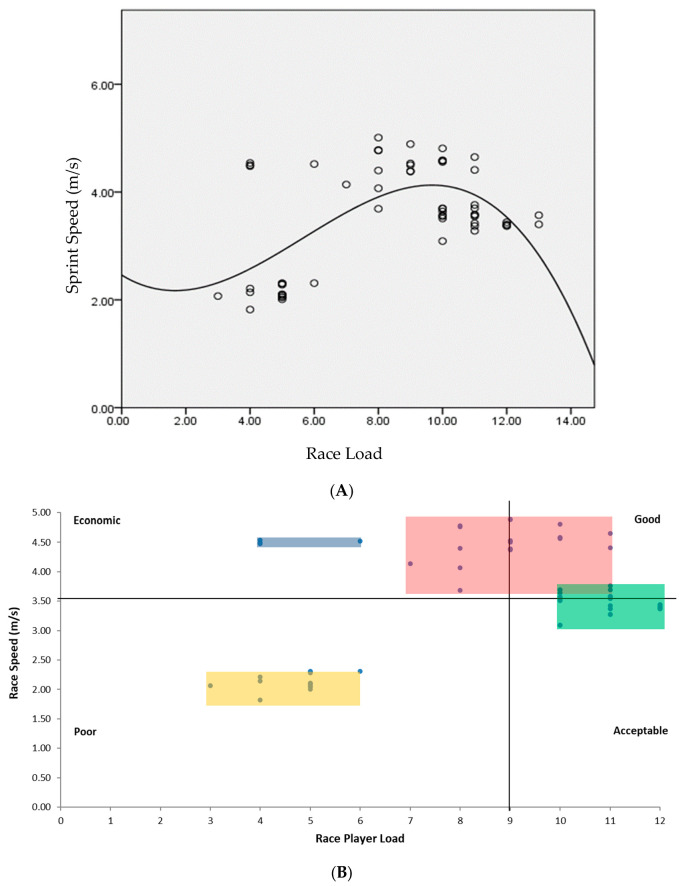
There was a non-linear relationship between race load and sprint performance in RaceRunners (**A**). This relationship was established as a criterion (significant association) to evaluate individual performance and for creating a classification system to evaluate training load (X), relative to sprint performance (Y). The vertical and horizontal lines are group median scores (**B**). The colours represent within class distributions.

The relationship between race load and sprint performance was statistically significant and non-linear (r = 0.61, R^2^ = 37%, *p* < 0.05), which indicates that the best sprint velocity was achieved in the race loads between 8 and 10 g. This association established a model to classify the performers. The long-term monitoring of sprint load and performance showed that the athletes differed in performance efficiency. In other words, the load–performance relationship emerged in four distinct groups, listed as economic, good, acceptable and poor performers (see Figure 4). These categories (quadrants) mainly emerged from the median scores of the group performance (all individual values) in different sessions based on race load (X) and running speed (Y) and neighbour points to create a cluster. Thus, athletes might move from one category (performance quality) to another one due to fluctuations in race load and race speed (see Figure 3).

### 3.2. Technical Adaptations

**Figure 5:**
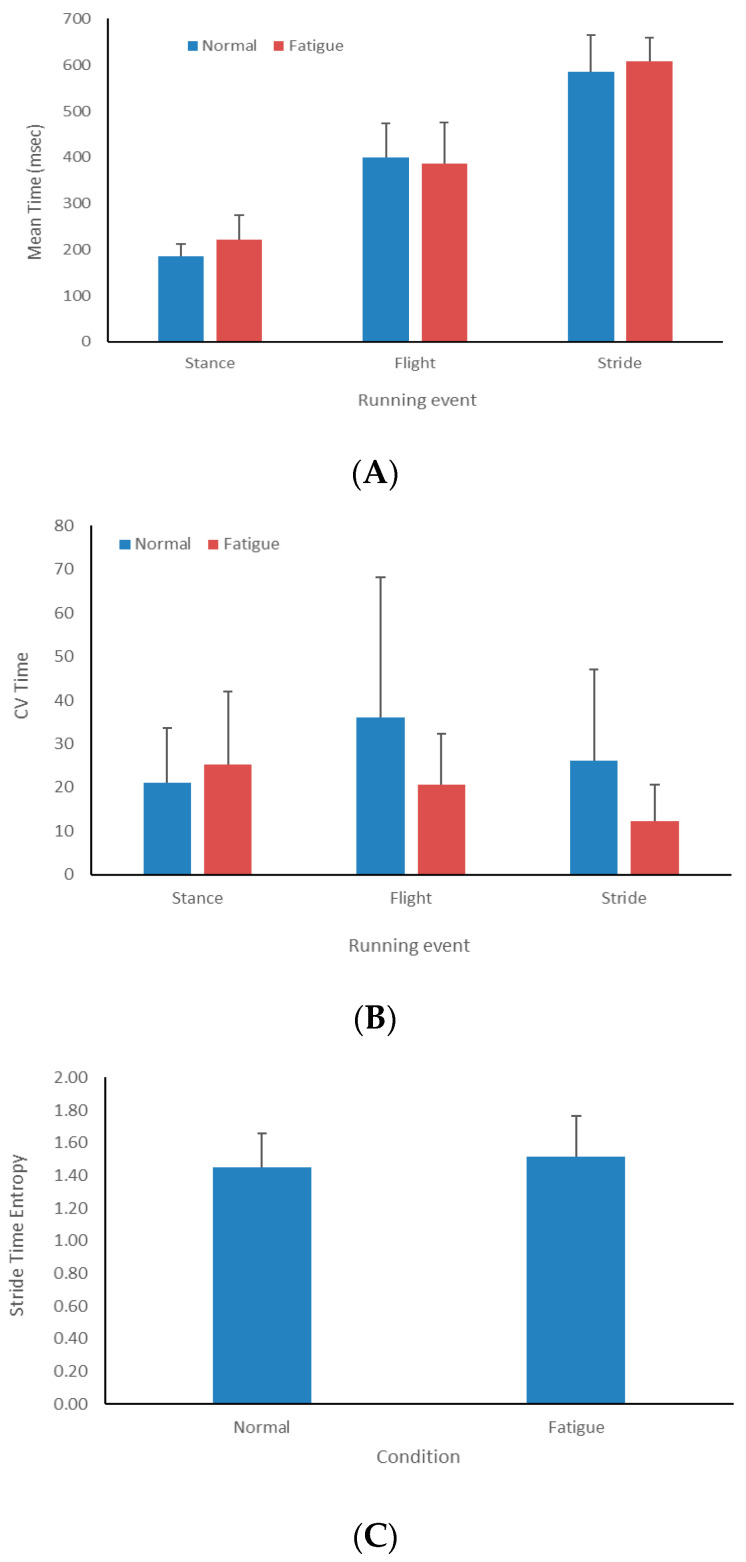
Running gait profile of RaceRunning athletes in different training conditions. Temporal mean (**A**), variability (**B**) and consistency (**C**) of running cycle are different between normal and fatigue conditions.

The wearable sensors were able to assess the temporal pattern of the running cycle during training sessions (see Figure 5). The majority of athletes spent more time in the stance phase when the training session progressed from the first race (normal condition) to the last race (fatigue condition), which was accompanied by increased temporal variability in the stance phase. The flight phase and stride times and variability were unchanged or decreased as the training session progressed. The stride time value was slightly less consistent when the training session progressed. However, the results of the Wilcoxon test did not show any statistically significant differences in running performance, variability and consistency between the two conditions (*p* > 0.05). The magnitudes of ES for the average time, temporal variability and temporal consistency were medium (d = 0.58), medium (d = 0.66) and small (d = 0.22), respectively.

**Figure 6:**
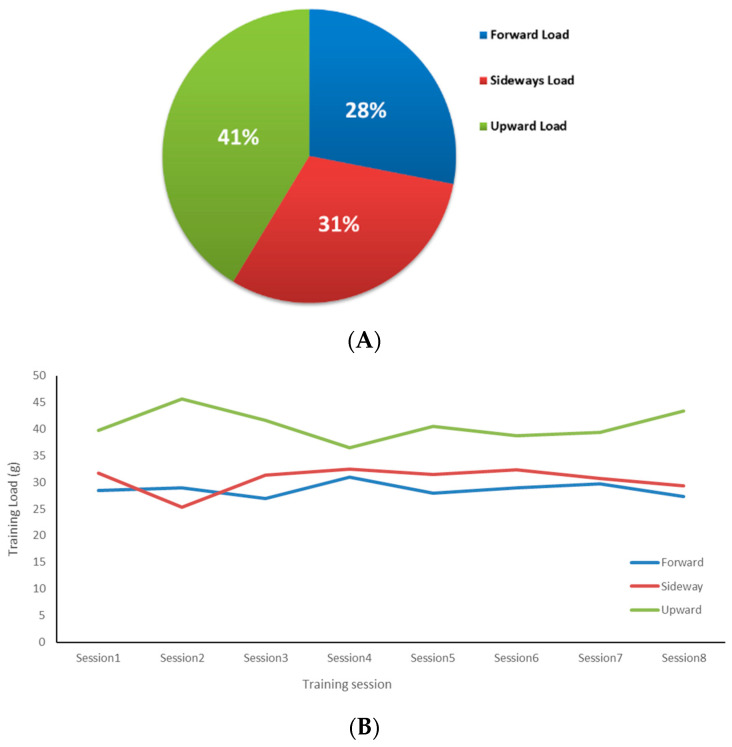
Variations in training load during the whole study period (**A**) and in every session (**B**). The athletes shared the external loads in different directions, with slightly variations between sessions.

Results of the Friedman test showed that the direction of external loads was significantly different (χ^2^ = 8.40, *p* < 0.05) and the majority of external load was transferred vertically (41%). The magnitude of ES was large (*d* = 2.33). The trends were similar in different training sessions, except in session 2, where the forward load was slightly higher than the sideways load (see Figure 6).

Applications of some performance indicators for training monitoring are summarised in Table 1.

## 4. Discussion

The aims of this study were to monitor training loads in typical training sessions and evaluate technical adaptations in RaceRunning performance from sensor metrics. The findings of the current study showed that feedback provided by a wearable-based system was useful in obtaining more information about the quality of training, such as (i) the individual’s adaptations to sprint performance as external loads increased, (ii) acceptability of weekly load changes to prevent overuse injuries and overtraining, and (iii), technical changes in running temporal parameters due to perceived fatigue at the end of training sessions. The advantages of the wearable-based system as a performance analysis tool for disabled athletes are discussed in the following sections.

### 4.1. Wearable Sensors for Monitoring Training Loads

The first application of wearable sensors was for monitoring training loads. The wearable motion sensors were feasible for in-field analysis of the performance of disabled athletes. The performance metrics in this study were important to investigate the adverse effects of training, especially in disabled runners who have limited capacity to adapt to the excessive training volume. In other words, metrics such as total sessions load (player loads), race load, activity/rest ratio and ACWR are conventional performance metrics in sport to monitor body adaptations and compensations. Two criteria have been used in monitoring and decision-making of the training loads: SWC and ACWR. Assessment of both criteria confirmed that the athlete’s response to the training loads was acceptable and the training did not cause any adverse effects. An important aspect of sport training concerns the body’s adaptations to the training stimulus. Therefore, the rate of change in training load may be more problematic than the absolute load experienced by an individual. Hence, ACWR is a useful metric to calculate an athlete’s ability to tolerate sudden changes in load [19]. This is the first study that used a wearable system in disabled athletes to monitor the training loads. However, in able-bodied distance runners [17], it has been reported that the normal range of ACWR was between 0.8 and 1.3, more than the normal range of ACWR reported in this study (0.52–0.79). Those participants also showed more weekly fluctuations in ACWR than in this study.

Determining whether weekly changes in training load are clinically and practically meaningful is also important in sport training [33]. The SWC is a criterion that can distinguish between random variations (individual and situational differences) and systematic variations (training adaptations). It should be used to understand the meaningfulness of any observed changes instead of changes based on cut-off points [34]. Calculating the SWC allows the coach to be confident that they can accurately determine a real change in body responses to the training over time, rather than just a typical variation. Our results showed that all athletes performed in the acceptable zone regardless of their disability status. Thus, matching between task demands (training volume) and individual constraints has been undertaken precisely in this study by using the wearable-based system, which was not possible with traditional methods of training assessment.

Based on the non-linear relationship between race loads and sprint velocity, four RaceRunning groups emerged: runners with economic, good, acceptable and poor performances. This finding is important in determining the level of physical fitness in more representative sport training. In other words, the majority of traditional sport analysis methods are based on recording training factors such as the number of races, time and distance rather than how the athletes tolerate the training stress [21]. This uni-dimensional approach (performance-oriented) overlooks the person–task interaction (bi-dimensional approach) in planning and might lead to overtraining and subsequent overuse injuries. Including wearable sensor-based training metrics (e.g., race loads) in addition to the conventional methods could help runners to individualise their training adaptations [22], in adopting a reasonable approach for progression from poor performance to economic performance. After inspection of individual performance (for example, ACWR in Figure 3C and the classification system in Figure 4A), it was evident that the level of inter-individual variability (indexed as SD bars and XY individual points) was a key factor in discriminating athletes. The method of feedback provision to individualise the training condition through a wearable-based system was more appropriate to meet the individual’s needs.

It seems that maintaining energy levels in successive sprint attempts in a training session after adequate rest periods was a key performance variable that distinguished the athletes of this study. As a result, it is not surprising that the performance–load association was non-linear and that the economic sprint performance had a race load between 8 and 10 g. Other individual factors that could be mediators of this relationship are the roles of power, technique and sprint-specific endurance in sprinting performance [35], as well as differences in pacing strategy (acceleration, maximal velocity and deceleration phases) between runners of this study [36]. These individual factors should be investigated in future studies to clarify the role of organismic (personal) constraints in the performance–training load relationship.

### 4.2. Wearable Sensors for Identifying Technical Adaptations

The second application of wearable sensors in this study was for finding possible modifications in the running gait cycle. The repeated sprints in a session showed that the athletes performed differently as the training session progressed from early to later attempts. The changes in temporal parameters had bigger impacts (ES) on cycle timing and variability than consistency. The variability and consistency of running cycle time have different meanings in biomechanical adaptations to training stress. Whilst the time of stride and each stride phase (stance and flight) indicate the speed of action execution, the other two metrics, variability and consistency, are related to motor control mechanisms and can provide more information about running performance. For example, driving a three-wheeled bike with existing disabilities requires significant postural and muscular adjustments in this population, and that might cause performance fluctuations (variability) in training sessions. We have to note that the participants of this study were competitive athletes with sufficient training experience in this sport, but their technical performance was still affected by training stress. This information could be used as a feedback tool to assess physical conditioning (e.g., fatigue threshold) through a more functional approach.

The scope of previous studies in RaceRunning is limited only to assessment and general fitness improvements [37,38], and there are no studies which shed insights on the athletes’ biomechanical adaptive capacities. However, the temporal modifications in sprint performance, due to training factors such as fatigue, are common in running. For example, García-Pinillos et al. [39] showed that fatigue increased stance time by 4%, step time variability by 5% and reduced flight time by 15% in sprint performance.

Another technical adaptation which was picked up in data from the wearable motion sensors was related to variations in the impact shocks. The results showed that vertical shock is stronger than shocks in other directions, and this trend was similar in different training sessions. Adaptations to the ground impact shock in every stride are a natural ability, but due to some technical differences such as foot placement, body sway and coordination with the bike, the amount of shock transfer in the 3D body plane might be variable. This finding showed that the postural control mechanisms work flexibly to stabilise the body and propel it forward in fast running performance, an ability that is developed by practice and experience. Other adaptive functions, such as an ability to attenuate body impact shock, have also been reported in RaceRunning athletes in a previous study [2].

Our study has practical implications for para sports generally, and RaceRunning specifically. It is not possible to provide kinematic feedback in a typical training session by using traditional methods, and usually, coaches only measure overall running time as a feedback metric. We showed that using wearable motion sensors provided additional information about the running gait cycle to monitor subtle changes in the temporal patterning of athletes due to individual (level of disability), environmental and task constraints (training factors). The performance metrics defined in this study have functional benefits in monitoring training loads and also gaining insights into the technical adaptations. One example of application in routine practice by coaches may be considered by interacting with the motion sensors dashboard that provides online and concurrent feedback on the screen during or after a race. Use of these data may be useful for comparing the results of external load and heart rate (maximum and average) with previous attempts (accumulated scores) to assess the training intensity and volume.

As a limitation of this study, calculating the running phase by mathematical algorithms in this study is not practical for in-field assessment, but advances in technologies and machine learning methods can lead to innovative ways to assess running gait adaptations during and after training in future. The other limitations of this study were relying on a small (convenience) sample size and skill level of performers that might restrict the generalisability of this study in disabled RaceRunning athletes. Incorporating the rate of perceived exertion (RPE) in the assessment system and associating with the heart rate and player loads is another limitation of this study that can be investigated in future research. Technically, wearing sensors during strenuous activities, such as sprint running, could be subject to measurement error due to losing sensor–skin contact specifically with the waist sensor. This issue might be negligible in individual sports such as running (where there is no physical contact). Using it in other activities might affect the reliability of data in prolonged sessions.

## 5. Conclusions

The findings of this study showed how using a wearable-based system could enhance the quantity and quality of augmented feedback over traditional methods of performance analysis in monitoring training loads and identifying technical adaptations in sprint performance in disabled athletes. The results suggest how coaches and performance analysts can use metrics such as training loads, race loads, impact shock directions and the load–performance quadrants as feedback tools to enhance running performance in disabled athletes. It is highly recommended that future research design studies based on the limitations of the current study to achieve better findings for practice changes, mainly through accessing a bigger sample size and using a more systematic approach in applying all performance criteria.

## Figures and Tables

**Table 1:** A summary of performance indicators for monitoring training in RaceRunning.

*Performance Indicator*	*Applications to Training Monitoring*
Training load	Assessing the runner and overall training physical stress through resultant XYZ acceleration
Race load	Assessing training physical stress in running events (e.g., 60 m, 100 m dash, etc.) through resultant XYZ acceleration
ACWR	Assessing the quality of an individual training physical stress (overstressed/normal) relative to monthly average training stress for prevention of overtraining and overuse injuries
Running temporal pattern	Assessing temporal features of running pattern and their changes in response to training stimulus (internal adaptations) through variability and regularity measures
Directions of external loads (impact shocks)	Assessing the load distribution patterns and their variations during running

## Data Availability

The data presented in this study are available on request from the corresponding author.
